# Interpretation of the post-surgical Somatostatin Receptor Scintigram of a Primary Neuroendocrine Tumor of the Thymus: a case report and literature review

**DOI:** 10.1186/1477-7800-2-7

**Published:** 2005-03-23

**Authors:** Anastasia Leondi, John Koutsikos, Cherry Zerva

**Affiliations:** 1 Department of Nuclear Medicine, Alexandra University Hospital, Athens, Greece

**Keywords:** mediastinum, neuroendocrine carcinoma, thymic carcinoid tumor, somatostatin receptor scintigraphy.

## Abstract

A case of a thymic neuroendocrine tumor and the interpretation problems in a post-surgical Somatostatin Receptor Scintigraphy are presented. In a 53-year-old man with superior vena cava obstruction syndrome an atypical carcinoid of the thymus (*neuroendocrine carcinoma of intermediate grade 2*), was found at surgery.

During his first year of follow-up a Somatostatin Receptor Scintigraphy was recommended. An area of abnormal concentration of the radiopharmaceutical was revealed in the mediastinum at this time.

A thorough understanding of the mechanisms of the radiopharmaceutical uptake and of the various clinical settings in which uptake can occur are essential for a proper evaluation of the scintigraphic findings and result in the optimal use of this valuable modality.

The literature review provides an overview of this rare type of tumor and insight into the clinical significance of Somatostatin Receptor Scintigraphy.

## Introduction

Primary neuroendocrine tumors of the thymus, previously known as thymic carcinoids, are unusual tumors that account for less than 5% of all anterior mediastinal neoplasms. They affect patients over a wide age range, with the median age being 43 years. Men are more frequently affected than women, with a male to female ratio of 3:1 [1].

Clinically, these tumors manifest in one of four ways: 1) as an incidental finding on routine chest radiography, 2) with symptoms of thoracic structure displacement or compression, 3) with symptoms related to an associated endocrinopathy or 4) with symptoms and signs relating to a distant metastasis, most commonly in the liver, lung, pancreas, pleura or bone. At least 20% of affected patients have metastatic disease at presentation, with the frequency of extra-thoracic metastasis being 20% – 30 % [2,3]. Approximately one-half of thymic neuroendocrine tumors (TNET) are functionally active and manifest with clinical hormone-excess syndromes, such as Cushing syndrome and are part of the autosomal dominant syndrome of multiple endocrine neoplasia (MEN) [4]. Type I (MEN) syndrome is characterized by hyperparathyroidism, islet cell tumors of the pancreas, and pituitary adenomas. Carcinoids, adrenal adenomas or carcinomas, lipomas and follicular thyroid adenomas are less frequently associated neoplasms. The majority of patients with TNET and type I MEN syndrome are male. Additional associated conditions found in patients with TNET include type 2 MEN syndrome, inappropriate secretion of antidiuretic hormone, polymyositis, finger clubbing, polyarthropathy, and myocarditis.

Thoracic CT and MRI and nuclear medicine imaging, including Somatostatin Receptor Scintigraphy (SRS), meta-iodobenzylguanidine (MIBG) scintigraphy with I-123 or I-131 and bone scan, are useful studies in the evaluation of these patients [5]. Radio labeled somatostatin receptor analogues can detect biochemical lesions. This constitutes an advanced option of "functional" imaging. The success of imaging a certain lesion depends on the somatostatin receptor subtypes (SSRT) that are expressed by the tumor. In-111-DTPA-D-Phe 1-pentetreotide (pentetreotide) has shown highest affinity for SSRT2, SSRT3 and SSRT5. Its total sensitivity in detecting neuroendocrine tumors is reported to be 71% – 100% depending on the histological type of the tumor. MIBG scan sensitivity has been reported to be 16% – 96% in these tumors [6]. Few reports of cross-sectional imaging features of TNET have been published; these lesions have been described as anterior mediastinal masses indistinguishable from thymomas at CT. Invasion of focal structures and calcification within the tumor have both been reported.

However, pentetreotide has also been shown to concentrate in primary and metastatic thymic tumors, including thymoma, thymic carcinoma, and TNET [7-9] where subtype SSRT2 is present in high density.

SRS also may have a role in the follow-up of these patients [9]. Another potential application of the pre-surgical SRS is its use as a guide for octreotide therapy [10] and for radionuclide therapy with radio labeled analogues under specific conditions and criteria. Systemic injection of ^111^In-Pentetreotide has yielded tumor growth inhibition in animal models and in patients with somatostatin receptor positive tumors, this therefore could be a clinical application in this kind of tumor [11-13].

### Case presentation

**Figure 1:**
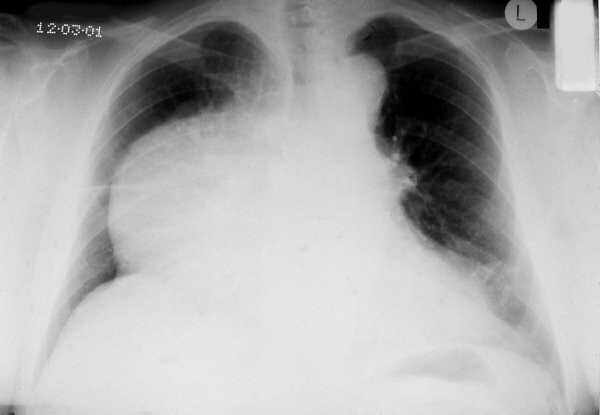
The chest x-ray, showing a large mass projecting over the right lung field.

**Figure 2:**
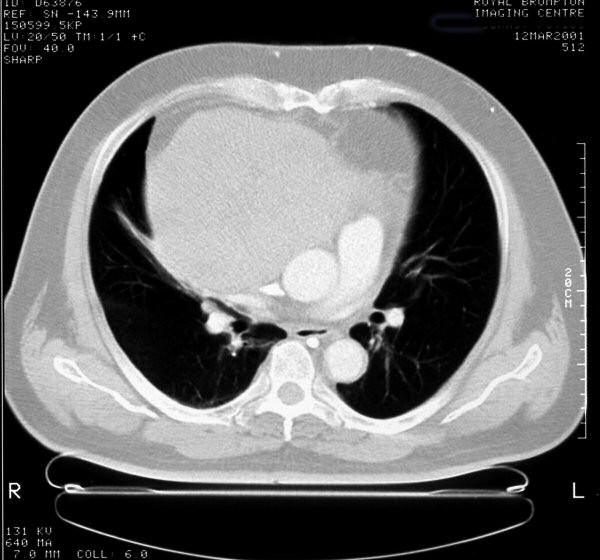
A pre-surgical CT scan of the chest showing the tumour.

A 53-year-old man noticed some swelling of his face. A chest x-ray showed a large mass projecting over the right lung field (fig 1). A CT scan of the chest showed an enormous anterior mediastinal mass contiguous with the posterior aspect of the sternum and the right ribs, occluding the superior vena cava and compressing the heart (fig 2). Blood tests were normal, tumor markers and Acetyl Choline Receptor Antibodies were negative. The patient underwent bronchoscopy and the biopsy showed the features of a carcinoid tumor.

He underwent excision of the tumor, which required resection of the superior vena cava and insertion of a graft, resection of the right phrenic nerve and plication of the diaphragm.

The finding at surgery was an enormous spherical tumor with an extremely vascular capsule due to the venous collaterals. The tumor was 20 cm from pole to pole and 15 cm in diameter. On the left it bulged into the left pleural space, displacing the phrenic nerve laterally. Superiorly it obliterated the left innominate vein and extend into the strap muscles. There was an enlarged lymph node. On the right it had obliterated the confluence of the innominate veins and the first centimeter or so of the superior vena cava. It enclosed the phrenic nerve over its complete length and was adherent to the medial aspects of the upper and middle lobes of the right lung. There was some thickening of the visceral pleura in this area and there appeared to be some superficial infiltration. Posteriorly it was adherent to the pericardium and there was some thickening of the ascending aorta, consistent with longstanding pressure effects. There was a small bloody pericardial effusion and a rather large right pleural effusion. There was no evidence of any pleural deposits or intrapericardial deposits.

The histology of the resection specimen showed a tumor weighing 2.1 kg. The tumor appeared encapsulated with an irregular surface. The tumor invaded the surrounding fat and pleura. There was infiltration into the underlying lung tissue lymphatics. There was some residual thymic tissue within the surrounding fat. Metastatic deposits were found in the right hilar lymph node and the node removed from the left cervical horn of the thymus. The tumor was classified as an atypical carcinoid of the thymus (**neuroendocrine carcinoma of intermediate grade 2**).

One month later he underwent a CT scan of the chest. There were findings consistent with a recent sternotomy and a graft of the superior vena cava. There was a fluid filled cyst at the base of the right lung and areas of atelectasis and pleural thickening were noted in both lungs.

An I-123 MIBG scan was performed with negative results for detection of any residual or recurrent pathology.

He was treated with postoperative radiotherapy and chemotherapy.

One year later, a follow-up CT scan of the chest was obtained with no findings of recurrence or abnormal lymph nodes in the mediastinum. Linear atelectasis was detected in the lower lobes of both lungs and elevation of the right hemi diaphragm was noted. In addition a bone scan was performed with a positive finding at the posterior arch of the 8^th ^right rib. The patient was advised to have a SRS, for a more accurate evaluation of his status, as it was considered that "functional" imaging performed by Nuclear Medicine examinations, might detect pathological processes earlier than "structural" images, such as CT.

Spot images of head-neck, thorax, abdomen and lumbar region were obtained 3 hr after injection of 5 mCi In-111-DTPA-D-Phe 1-pentetreotide. The scan was carried out on a Sopha single headed tomographic gamma camera coupled to a dedicated NXT computer system. Spot images of the areas of interest were obtained with a high-energy all-purpose collimator using 20% windows centered at 171 and 245 keV.

**Figure 3:**
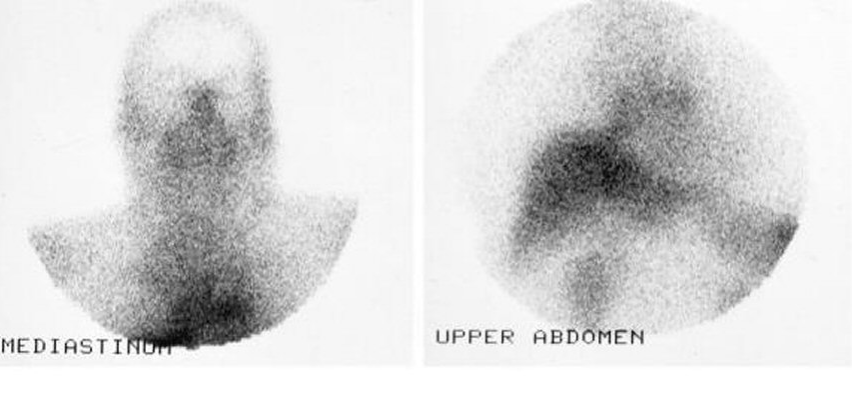
SRS with In-111-DTPA-D-Phe 1-pentetreotide showed an area of abnormally increased concentration of the radiopharmaceutical at the anatomical area of the mediastinum.

The SRS was reported as showing an area of abnormally increased concentration of the radiopharmaceutical at the anatomical area of the mediastinum due to either a mass remnant or a recurrence of the disease or an inflammatory process (fig. 3). The bone scan finding did not take up the radiopharmaceutical. A repeat bone scan after a further 9 months was negative.

Thus, the main questions that we had to answer were: is there a mass remnant, or a recurrence of the disease? and, can we exclude a metastatic lesion at the site of the finding on the bone scan?

## Discussion

Although there was no pre-operative SRS, requesting a post-operative SRS for this patient was justified because of the histological type of his tumor, the extensive lesions and the extended and complicated surgical management. Additionally, it is possible that during surgical maneuvers some cells were transported and implanted in other organs, so that post-surgical metastasis had occurred.

Another serious reason for referring such a patient for a SRS is the finding on the bone scan [14]. Regional lymph node and distant metastases, including osteoblastic bone metastases, have been reported in up to 73% of cases and can occur late. An accidental injury could also be detected by the bone scan and for a period of 1 year. In the SRS, no lesion was found compatible with the finding in the bone scan. This, together with the fact that the bone scan lesion was solitary, reduces the probability of a metastatic bone lesion, though the possibility of variation of expression of SSRTs between metastases and primary tumor remains. This explains, in part, why the MRI scan is reported to be a more sensitive method than SRS [5].

The patient underwent a post-surgical ^123^I MIBG scan that was negative. The ^123^I MIBG scan was also performed in order to detect neuroendocrine tissues, but its sensitivity is unknown in this tumor. We note that SRS detects primary and metastatic thymic tumors, including TNET [7,8,14].

As to the interpretation we gave to the SRS, this could not be more precise in answering whether there is a mass remnant or a recurrence of the disease, for the following reasons:

1. There wasn't a pre-surgical SRS for comparison. So it was not known whether or not the primary mass could be imaged with pentetreotide. A negative scan may result when the SSRTs of the tumor are different from those detected by the radiopharmaceutical.

2. There have been extended and complicated surgical maneuvers that may have cause anatomical changes.

3. An inflammatory process may exist in the same area which could also cause accumulation of the radiopharmaceutical.

This inflammatory component may be due to post radiotherapy fibrosis or to co-existing inflammatory reaction in the atelectasis of the lungs, because of the alveolar wall thickening and fibrosis.

The atelectasis, may have been caused by the surgical maneuvers but it is known that linear or disc-like atelectasis is common in the inferior lung lobes secondary to pleural, pericardial or mediastinal lesions, as was present in our patient.

It has also been recorded that if an atelectatic lesion has not resolved after three months, it will not return to normal tissue and constitutes a favourable site for mild infections (bronchectasis and pulmonary fibrosis). In these cases total blood count and ESR remain normal.

Pentetreotide is accumulated in surgical trauma because of the healing process in which macrophages are recruited. This fact makes a SRS unable to distinguish the disease from the healing process. This applies for two months after the surgery.

In the case of our patient, the SRS was performed one year after the surgery, so this possibility should be excluded, although the radiotherapy, which our patient had undergone, may have prolonged the healing period.

Two years after the SRS, the patient is free of disease according to his clinical condition, biochemical data and imaging modalities, including a MRI scan. A second SRS that could clarify our finding was not performed since the MRI scan was negative, and a further study of our finding was not possible. Thus, the SRS finding has to be interpreted, based on the above follow up data, as a radiopharmaceutical accumulation at the site of an inflammatory process and not as a mass remnant or a recurrence of the disease.

To our best knowledge, 28 cases of TNET in which SRS was performed have been reported to date in the literature (table 1) [5,7-10,15-18], and only one study in abstract form has reported a patient with TNET with MEN 1 having a "false" negative SRS [17]. Tiffet et al. [19] in a study of 12 neuroendocrine tumors arising in the thymus, found that none of the 12 tumors stained positively for somatostatin receptors, while Boix et al. in a case of a 33 year-old-woman with MEN 1 described positive somatostatin receptor staining by immunohistochemistry [20]. From the first study, it would seem that SRS is not advisable for post-operative follow up, while from the second, a post-operative SRS could be performed even if pre-operative SRS was not done.

**Table 1:** Series of Primary Neuroendocrine Tumors (PNET) of the Thymus, studied by Somatostatin Receptors Scintigraphy (SRS) with ^111^In-DTPA-D-Phe 1-pentetreotide.

**Author**	**Year**	**No of cases**	**Results**
Zahner et al [15]	1994	1	Positive
Cadigan et al [7]	1995	1	Positive
Teh et al [16]	1998	3	Positive
Lastoria et al [8]	1998	3	Positive
Satta et al [9]	1999	2	Positive
Grimfjard et al [17]	2002	3	2 positive/1 Negative
Gibril et al [5]*	2003	8	Positive
Plachcinska et al [18]**	2004	1	Positive
Loehrer et al [10]	2004	6	Positive

* 5 cases with initial and 3 with recurrent thymic carcinoid

** SRS with ^99 m^Tc-EDDA/HYNIC-TOC

## Conclusion

Since CT and MRI are more accurate than SRS in the detection of TNET, the main role of pre-operative SRS is in the planning of follow up and possible therapeutic approaches for these patients. The post-operative SRS should be performed at the right time, so that the healing process is over and a possible remnant or recurrence can be detected. All the radiopharmaceutical accumulation mechanisms have to be taken into account in order to proceed to a valid differential diagnosis and give precise information to the clinician.

## Competing interests

The author(s) declare that they have no competing interests.
